# Intratumoral peptide injection enhances tumor cell antigenicity recognized by cytotoxic T lymphocytes: a potential option for improvement in antigen-specific cancer immunotherapy

**DOI:** 10.1007/s00262-012-1366-6

**Published:** 2012-11-11

**Authors:** Daisuke Nobuoka, Toshiaki Yoshikawa, Mari Takahashi, Tatsuaki Iwama, Kazutaka Horie, Manami Shimomura, Shiro Suzuki, Noriko Sakemura, Munehide Nakatsugawa, Hiroshi Sadamori, Takahito Yagi, Toshiyoshi Fujiwara, Tetsuya Nakatsura

**Affiliations:** 1 Division of Cancer Immunotherapy, Research Center for Innovative Oncology, National Cancer Center Hospital East, 6-5-1 Kashiwanoha, Kashiwa, 277-8577 Japan; 2 Department of Gastroenterological Surgery, Okayama University Graduate School of Medicine, Dentistry, and Pharmaceutical Sciences, 2-5-1 Shikata-cho, Kita-ku, Okayama, 700-8558 Japan

**Keywords:** Intratumoral peptide injection, Antigen, Immunotherapy, Cytotoxic T lymphocyte

## Abstract

Antigen-specific cancer immunotherapy is a promising strategy for improving cancer treatment. Recently, many tumor-associated antigens and their epitopes recognized by cytotoxic T lymphocytes (CTLs) have been identified. However, the density of endogenously presented antigen-derived peptides on tumor cells is generally sparse, resulting in the inability of antigen-specific CTLs to work effectively. We hypothesize that increasing the density of an antigen-derived peptide would enhance antigen-specific cancer immunotherapy. Here, we demonstrated that intratumoral peptide injection leads to additional peptide loading onto major histocompatibility complex class I molecules of tumor cells, enhancing tumor cell recognition by antigen-specific CTLs. In in vitro studies, human leukocyte antigen (HLA)-A*02:01-restricted glypican-3_144–152_ (FVGEFFTDV) and cytomegalovirus_495–503_ (NLVPMVATV) peptide-specific CTLs showed strong activity against all peptide-pulsed cell lines, regardless of whether the tumor cells expressed the antigen. In in vivo studies using immunodeficient mice, glypican-3_144–152_ and cytomegalovirus_495–503_ peptides injected into a solid mass were loaded onto HLA class I molecules of tumor cells. In a peptide vaccine model and an adoptive cell transfer model using C57BL/6 mice, intratumoral injection of ovalbumin_257–264_ peptide (SIINFEKL) was effective for tumor growth inhibition and survival against ovalbumin-negative tumors without adverse reactions. Moreover, we demonstrated an antigen-spreading effect that occurred after intratumoral peptide injection. Intratumoral peptide injection enhances tumor cell antigenicity and may be a useful option for improvement in antigen-specific cancer immunotherapy against solid tumors.

## Introduction

Conventional modalities of cancer treatment, including surgery, radiotherapy, and chemotherapy, have made advancements in recent years, and the survival rate of cancer patients has gradually improved; however, these therapies remain far from being satisfactory in most cancers [1, 2]. Therefore, the development of novel treatment modalities, including antigen-specific cancer immunotherapies with peptide vaccines, dendritic cell vaccines, and adoptive cell transfer therapies, is critical for advancing effective cancer treatments [3–5]. While many tumor-associated antigens and epitopes recognized by cytotoxic T lymphocytes (CTLs) have been explored as possible antigen-specific cancer immunotherapies, the results of several anticancer immunotherapy clinical trials have been disappointing [6, 7]. We conducted a clinical trial using the glypican-3 (GPC3) peptide vaccine in advanced hepatocellular carcinoma (HCC) patients. While this carcinoembryonic antigen overexpressed in HCC seemed to be an ideal target for anticancer immunotherapy [8–15], only immunological efficacy was apparent [16], whereas the clinical benefit was limited in patients [17]. Therefore, the establishment of an innovative strategy to link the antitumor immune response with the clinical response and to enhance the power of antigen-specific cancer immunotherapy is urgently required.

In the antigen-specific cancer immunotherapy concept, antigen-specific CTLs recognize and destroy tumor cells that present antigen-derived peptides using cell surface major histocompatibility complex (MHC) class I molecules. However, the density of the antigen-derived peptide endogenously presented on tumor cells is generally low, resulting in the ineffectiveness of antigen-specific CTLs [18]. This low density of presented antigen is one reason why antigen-specific cancer immunotherapy has been ineffective in clinical settings. One solution for overcoming this critical problem is to induce high-avidity CTLs. Such CTLs can recognize a smaller number of peptide–MHC class I complexes and would contribute to a better outcome [19]. Another solution is to enhance tumor cell antigenicity by means of additional peptide loading onto MHC class I molecules. Increasing the density of antigen-derived peptide would facilitate CTL recognition and destruction of the tumor cells.

In this study, we investigated whether intratumoral peptide injection would induce additional peptide loading onto tumor cells, and, if so, whether increased presentation would enhance antigen-specific CTL tumor cell recognition. Moreover, we evaluated whether intratumoral peptide injection could be a useful option for improvement in antigen-specific cancer immunotherapy against solid tumors.

## Materials and methods

### Synthetic peptides

The peptides used in this study were as follows: human leukocyte antigen (HLA)-A*02:01-restricted GPC3_144–152_ (FVGEFFTDV) peptide (American Peptide Company, Sunnyvale, CA), HLA-A*24:02-restricted GPC3_298–306_ (EYILSLEEL) peptide (American Peptide Company), HLA-A*02:01-restricted cytomegalovirus (CMV)_495–503_ (NLVPMVATV) peptide (ProImmune, Rhinebeck, NY, USA), HLA-A*24:02-restricted CMV_341–349_ (QYDPVAALF) peptide (ProImmune), HLA-A*02:01-restricted human immunodeficiency virus (HIV)_77–85_ (SLYNTYATL) peptide (ProImmune), and H-2 K^b^-restricted ovalbumin (OVA)_257–264_ (SIINFEKL) peptide (AnaSpec, Fremont, CA, USA). The peptides were dissolved and diluted in 7 % NaHCO_3_.

### Cell lines

T2 cells (HLA-A*02:01), which lack the transporter associated with antigen processing (TAP), were purchased from Riken Cell Bank (Tsukuba, Japan). The human liver cancer cell line HepG2 (GPC3^+^, HLA-A*02:01/A*24:02) was purchased from American Type Culture Collection (Manassas, VA, USA). The human liver cancer cell line SK-Hep-1 (GPC3^−^, HLA-A*02:01/A*24:02), human colon cancer cell line SW620 (GPC3^−^, HLA-A*02:01/A*24:02), murine lymphoma cell line RMA (OVA^−^, H-2 K^b^), EL4 (OVA^−^, H-2 K^b^), and EG7 (OVA^+^, H-2 K^b^) were kindly provided by Dr. Yasuharu Nishimura (Kumamoto University, Kumamoto, Japan). SK-Hep-1/GPC3 is an established stable GPC3-expressing cell line transfected with a human GPC3 gene, and SK-Hep-1/vec is an established counterpart cell line, in which an empty vector was transfected. EG7 cells are OVA-transfected EL4 cells. Cells were cultured at 37 °C in RPMI 1640 or DMEM medium (Sigma-Aldrich, St. Louis, MO, USA) supplemented with 10 % fetal bovine serum (FBS), 100 U/ml penicillin, and 100 μg/ml streptomycin in a humidified atmosphere containing 5 % CO_2_.

### Mice

Female BALB/c nude, NOD/SCID, and C57BL/6 mice (6–8 weeks old) were purchased from Japan Charles River Laboratories (Yokohama, Japan). OT-I mice [20], which are CD8^+^ T-cell TCR transgenic mice expressing the TCR α-chain recognizing OVA_257–264_ peptide in H-2 K^b^, were kindly provided by Dr. Takashi Nishimura (Hokkaido University, Sapporo, Japan). All animal procedures were performed according to the guidelines for the Animal Research Committee of the National Cancer Center, Japan.

### Preparation of OT-I mouse-derived CD8^+^ CTLs (activated OT-I CTLs)

Naïve CD8^+^ T-cells were purified from the spleens of OT-I mice using MACS anti-CD8a (Ly-2) MicroBeads (Miltenyi Biotec, Bergisch Gladbach, Germany). For in vitro activation, naïve OT-I CD8^+^ T-cells were incubated with irradiated EG7 cells at a 3:2 ratio in 24-well plates for 3 days. Each well contained 2.4 × 10^6^ OT-I CD8^+^ T-cells plus 1.6 × 10^6^ EG7 cells in 2 ml of RPMI 1640 medium supplemented with 10 % FBS, penicillin, streptomycin, and 50 μmol/l 2-mercaptoethanol. Activated OT-I CD8^+^ T-cells were separated from EG7 cells using anti-CD8a magnetic beads before adoptive transfer.

### IFN-γ ELISPOT assay

The BD™ ELISPOT set (BD Biosciences, San Jose, CA, USA) was used for an interferon (IFN)-γ enzyme-linked immunospot (ELISPOT) assay. CTLs were used as effector cells, and tumor cell lines with or without a peptide pulse (10 μg/ml for 1 h) were used as target cells. Effector cells (1 × 10^3^/well) were incubated with target cells (1 × 10^4^/well) in 200 μl of RPMI 1640 medium supplemented with 10 % FBS, penicillin, and streptomycin for 20 h at 37 °C in 5 % CO_2_. The number of spots, indicating an antigen-specific CTL response, was automatically counted using the Eliphoto system (Minerva Tech, Tokyo, Japan).

### Cytotoxicity assay

The Terascan VPC system (Minerva Tech) was used for cytotoxicity assays. Target cells were labeled with Calcein-AM (Dojindo Laboratories, Kumamoto, Japan) solution for 30 min at 37 °C, washed three times, distributed to 96-well culture plates in duplicate, and incubated with effector cells for 4 h. Fluorescence intensity was measured before and after the 4-h culture, and antigen-specific cytotoxic activity was calculated as described previously [16].

### Intratumoral peptide injection

In in vivo studies, tumors implanted on the backs of mice were injected with 50 μg peptide mixed with an equal volume of incomplete Freund’s adjuvant (IFA, Montanide ISA-51VG; SEPPIC, Paris, France). The total volume of solution injected was 100 μl in all experiments.

### Tumor excision and isolation of tumor cells

To investigate whether the injected peptide was loaded onto HLA class I molecules of tumor cells in a solid mass, an IFN-γ ELISPOT assay was performed using these isolated tumor cells as target cells. Mice were killed and their dorsal tumors were dissected, cut into small pieces, and digested with collagenase (1.5 mg/ml) for 20 min at 37 °C.

### In vivo tumor growth inhibition assay

In a peptide vaccine model, H-2 K^b^-restricted OVA_257–264_ peptide emulsified with IFA (50 μg/100 μl) was intradermally injected at the base of the tail of C57BL/6 mice, five times at 7-day intervals as described previously [13]. After vaccination, the induction of H-2 K^b^-restricted OVA_257–264_ peptide-specific CTLs was detected by IFN-γ ELISPOT assay (data not shown). In an adoptive transfer model, activated OT-I CTL (1 × 10^7^ cells/500 μl) was intravenously injected.

SW620 cells (5 × 10^6^ cells/100 μl) were subcutaneously implanted into the backs of BALB/c nude mice; SK-Hep-1/vec, SK-Hep-1/GPC3, or HepG2 cells (5 × 10^6^ cells/100 μl) were implanted into NOD/SCID mice, and RMA cells (5 × 10^4^ or 5 × 10^5^ cells/100 μl) were implanted into C57BL/6 mice. Tumor volume was monitored twice a week and calculated using the following formula: tumor volume (mm^3^) = *a* × *b* ^*2*^ × 0.5, where *a* is the longest diameter, *b* is the shortest diameter, and 0.5 is a constant to calculate the volume of an ellipsoid. Mortality and morbidity were checked daily, and the mice were maintained until each mouse showed signs of morbidity or the length or width of the tumors exceeded 30 mm, at which point they were killed for reasons of animal welfare.

### Tetramer staining and flow cytometry analysis

For the analysis of local accumulation of antigen-specific CTLs, isolated tumor cells, including tumor-infiltrating lymphocytes, were stained with H-2 K^b^ OVA Tetramer-PE (OVA_257–264_ [SIINFEKL]; MBL, Nagoya, Japan) for 20 min at room temperature and anti-mouse CD8-FITC (rat monoclonal, clone KT15; MBL) for 20 min at 4 °C. Flow cytometry analysis was carried out using a FACSCanto II flow cytometer (BD Biosciences).

### Immunohistochemistry

To investigate whether CD8^+^ T-cells infiltrated normal tissues due to intratumoral peptide injection in a murine adoptive cell transfer model, we performed immunohistochemical staining of CD8 in tissue specimens from C57BL/6 mice using monoclonal anti-CD8 antibody (dilution 1:20, BioLegend, San Diego, CA, USA).

### Statistical analysis

Comparisons of spot numbers and tumor volume at the last time point were performed using the Mann–Whitney U test. Survival was analyzed according to the Kaplan–Meier estimate, and differences between groups were compared using the log-rank test. Differences were considered significant at *P* < 0.05. Data were analyzed with the statistical package, Dr. SPSS II (SPSS Japan, Tokyo, Japan).

## Results

### In vitro CTL activity against peptide-pulsed targets

To evaluate the antigen-specific CTL response in vitro, IFN-γ ELISPOT and cytotoxicity assays were performed. In both assays, the two types of effector cells were the HLA-A*02:01-restricted GPC3_144–152_ peptide-specific CTL clone, which was established from peripheral blood mononuclear cells (PBMCs) of an HCC patient who had received the GPC3_144–152_ peptide vaccine [16], and the HLA-A*02:01-restricted CMV_495–503_ peptide-specific CTL clone, which was established from PBMCs of a healthy volunteer. The target cells were tumor cell lines with or without antigenic peptide pulses.

**Fig. 1:**
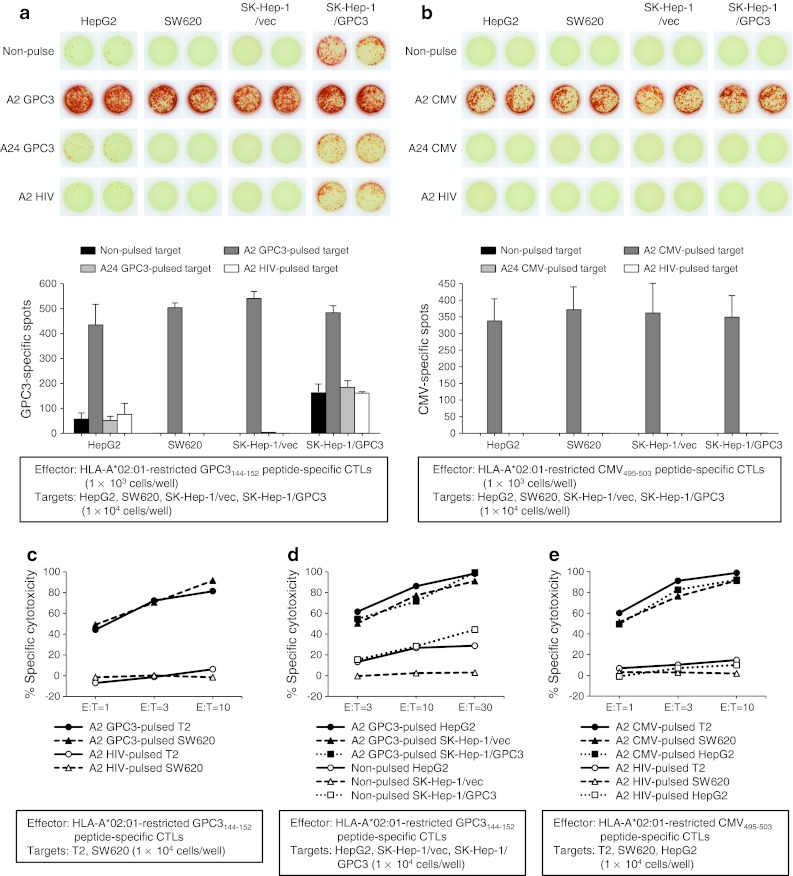
In vitro CTL activity against the peptide-pulsed targets. (**a** and **b**) IFN-γ ELISPOT assay. (**c**, **d**, and **e**) Cytotoxicity assay. HLA-A*02:01-restricted GPC3_144–152_ peptide-specific CTLs (**a**, **c**, and **d**) and HLA-A*02:01-restricted CMV_495–503_ peptide-specific CTLs (**b** and **e**) showed activity depending on the peptide density of tumor cells. Data are representative of three independent experiments, and bar graphs represent mean values of three independent experiments (SD) in (**a** and **b**)

As shown in Fig. 1a, in an IFN-γ ELISPOT assay, the HLA-A*02:01-restricted GPC3_144–152_ peptide-specific CTLs produced IFN-γ in the presence of GPC3-expressing tumor cells, HepG2 and SK-Hep-1/GPC3, without peptide pulse. These effector cells recognized GPC3_144–152_ antigen peptide, which is endogenously presented on the cell surface of the non-peptide-pulsed target cells. The number of IFN-γ-producing cells increased dramatically after the pulse of HLA-A*02:01-restricted GPC3_144–152_ peptide. In contrast, GPC3_144–152_ peptide-specific CTLs did not produce IFN-γ against GPC3-negative tumor cells, SW620 and SK-Hep-1/vec, without peptide pulse. However, a marked increase in IFN-γ-producing cells was detected against these cell lines after the pulse of HLA-A*02:01-restricted GPC3_144–152_ peptide. The IFN-γ-producing cells did not increase after the pulse of HLA-A*24:02-restricted GPC3_298–306_ or HLA-A*02:01-restricted HIV_77–85_ peptide (Fig. 1a). Similarly, HLA-A*02:01-restricted CMV_495–503_ peptide-specific CTLs produced IFN-γ only in the presence of HLA-A*02:01-restricted CMV_495–503_ peptide-pulsed target cells (Fig. 1b).

In a cytotoxicity assay, HLA-A*02:01-restricted GPC3_144–152_ and CMV_495–503_ peptide-specific CTLs showed antigen-specific killing activity according to the peptide density on tumor cells. HLA-A*02:01-restricted GPC3_144–152_ peptide-specific CTLs showed specific cytotoxicity against HLA-A*02:01-restricted GPC3_144–152_ peptide-pulsed SW620 and T2 targets, whereas they did not show cytotoxicity against HLA-A*02:01-restricted HIV_77–85_ peptide-pulsed targets (Fig. 1c). In addition, HLA-A*02:01-restricted GPC3_144–152_ peptide-specific CTLs showed apparent but weak cytotoxicity (13–44 %) against non-peptide-pulsed HepG2 and SK-Hep-1/GPC3 cells, but the cytotoxicity was markedly strengthened (55–99 %) against all examined cell lines after the HLA-A*02:01-restricted GPC3_144–152_ peptide pulse (Fig. 1d). Similarly, HLA-A*02:01-restricted CMV_495–503_ peptide-specific CTLs showed CMV_495–503_ peptide-specific cytotoxicity against all examined cell lines pulsed with CMV_495–503_ peptide (Fig. 1e).

The peptide-specific CTLs showed strong activity against all peptide-pulsed cell lines, regardless of whether the tumor cells expressed the antigen. The density of the HLA-A*02:01-restricted GPC3_144–152_ peptide endogenously presented on tumor cells was not enough to induce strong CTL activity.

### Loading of injected peptide onto HLA class I molecules of tumor cells in vivo

**Fig. 2:**
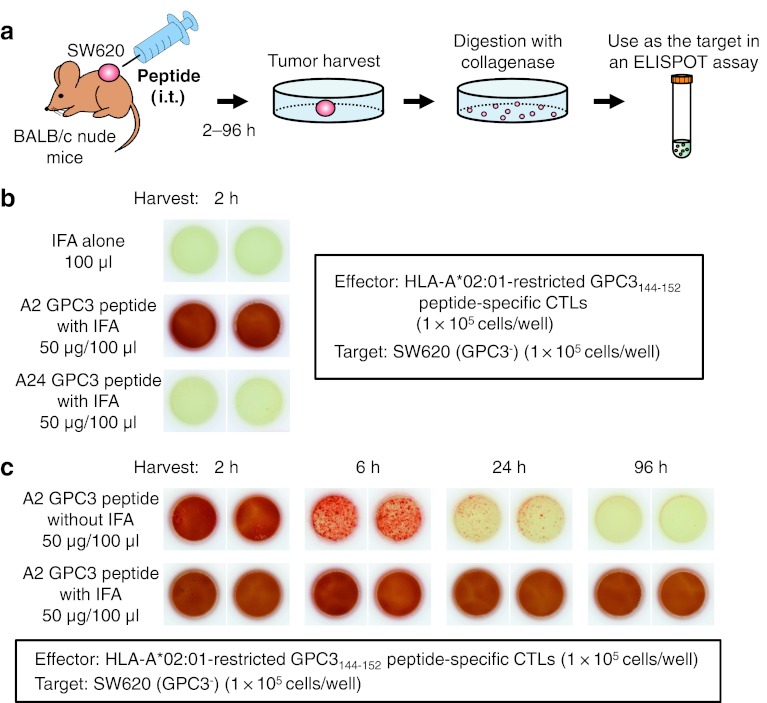
IFN-γ ELISPOT assay for loading of injected peptide onto HLA class I molecules of tumor cells in vivo. **a** Experimental schematic representation. **b** HLA-A*02:01-restricted GPC3_144–152_ or -A*24:02-restricted GPC3_298–306_ peptide emulsified with IFA was intratumorally injected, and the tumors were harvested after 2 h. IFA alone: no antigenic peptide; 50 μl of 7 % NaHCO_3_ was mixed with an equal volume of IFA. **c** HLA-A*02:01-restricted GPC3_144–152_ peptide with or without IFA was injected, and tumors were harvested at various times. Data are representative of three independent experiments

As shown in Fig. 2a, BALB/c nude mice were inoculated subcutaneously on their backs with SW620 (GPC3^−^) tumor cells. When tumor diameters reached 5–7 mm, 50 μg HLA-A*02:01-restricted GPC3_144–152_ peptide was injected into the tumor. After 2–96 h, the tumors were dissected, cut into small pieces, and digested with collagenase (1.5 mg/ml) for 20 min at 37 °C. To investigate whether the injected HLA-A*02:01-restricted GPC3_144–152_ peptide was loaded onto HLA class I molecules of tumor cells in a solid mass, an IFN-γ ELISPOT assay was performed in duplicate using these isolated tumor cells as target cells and HLA-A*02:01-restricted GPC3_144–152_ peptide-specific CTLs as effector cells.

Loading of HLA-A*02:01-restricted GPC3_144–152_ peptide onto HLA class I of tumor cells was detected (Fig. 2b). Without IFA, the density of loaded peptide gradually decreased after intratumoral peptide injection, whereas the loaded peptide density remained for 96 h after injection with IFA, suggesting that IFA is required for long-term stability of the injected peptide (Fig. 2c). Similar data were obtained with a combination of the HLA-A*02:01-restricted CMV_495–503_ peptide and its specific CTLs (data not shown).

### Antitumor effect of intratumoral peptide injection in an immunodeficient mouse model

**Fig. 3:**
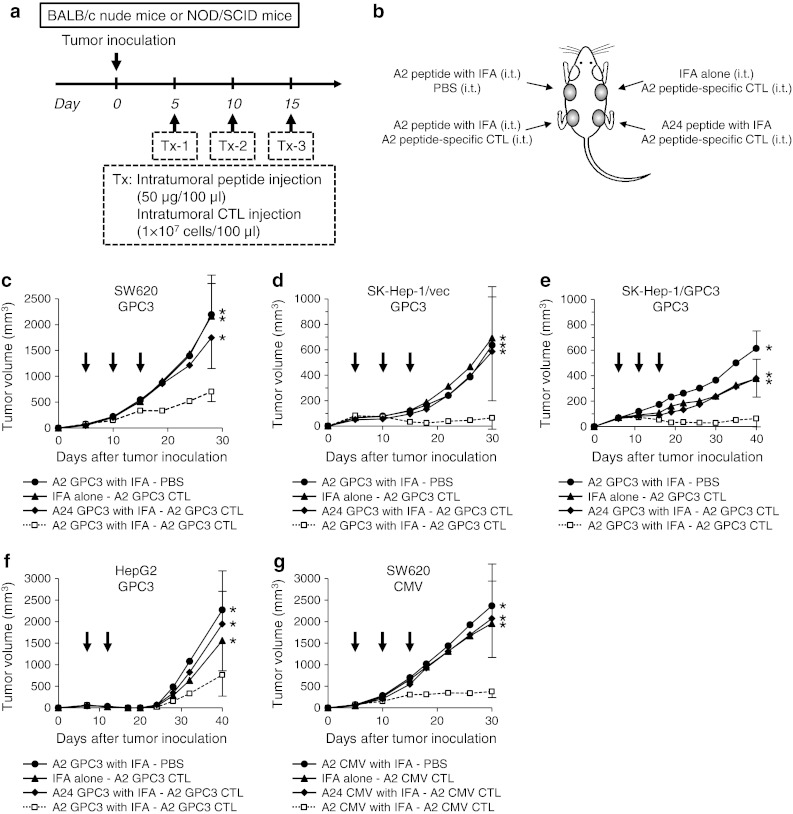
Antitumor effect of intratumoral peptide injection in an immunodeficient mouse model. Intratumoral injection of a combination of antigen peptide and its specific CTLs had a significant antitumor effect. **a** Treatment schedule. **b** Experimental schematic representation. BALB/c nude mice or NOD-SCID mice were inoculated subcutaneously on their *back* with SW620, SK-Hep-1/vec, SK-Hep-1/GPC3, or HepG2 tumor cells. Four tumors were implanted per mouse, and HLA-A*02:01-restricted GPC3_144–152_ or CMV_495–503_ peptide emulsified with IFA (50 μg/100 μl) and HLA-A*02:01-restricted GPC3_144–152_ or CMV_495–503_ peptide-specific human CTLs (1 × 10^7^ cells/100 μl) were injected into each tumor. (**c**, **d**, **e**, **f**, and **g**) Tumor volume. Tumor growth was expressed by mean tumor volume; *bars* (SD). Seven mice were used in each experiment. *Arrows* indicate the days when treatment was performed. *P < 0.05 compared with treatment group (Mann–Whitney U test)

We planned and executed the experimental schedule shown in Fig. 3a. Four tumors were implanted per mouse, and each tumor received a different combination of injections, as shown in Fig. 3b. From 5–7 days after tumor inoculation, mice were treated two or three times in 5-day intervals. The treatment regime was as follows: HLA-A*02:01-restricted GPC3_144–152_ or CMV_495–503_ peptide emulsified with IFA (50 μg/100 μl) was injected into a tumor, and, 2 h later, HLA-A*02:01-restricted GPC3_144–152_ or CMV_495–503_ peptide-specific human CTLs (1 × 10^7^ cells/100 μl) were injected into the tumor.

Intratumoral injection of a combination of HLA-A*02:01-restricted GPC3_144–152_ peptide and its specific CTLs resulted in statistically significant tumor growth inhibition (*P* < 0.05) (Fig. 3c). Similarly, this treatment was effective against SK-Hep-1/vec (Fig. 3d), SK-Hep-1/GPC3 (Fig. 3e), and HepG2 (Fig. 3f) tumors. Intratumoral injection of HLA-A*02:01-restricted GPC3_144–152_ peptide-specific CTLs alone against GPC3-expressing tumors, SK-Hep-1/GPC3 and HepG2, was only partially effective, suggesting that the HLA-A*02:01-restricted GPC3_144–152_ peptide endogenously presented on SK-Hep-1/GPC3 and HepG2 tumor cells was not sufficiently dense. However, intratumoral injection of HLA-A*02:01-restricted GPC3_144–152_ peptide increased the peptide density and markedly enhanced CTL activity. Similarly, intratumoral injection of HLA-A*02:01-restricted CMV_495–503_ peptide followed by its specific CTLs resulted in statistically significant tumor growth inhibition (*P* < 0.05) (Fig. 3g). Intratumoral injection of a combination of antigen peptide and its specific CTLs had a significant antitumor effect.

### Therapeutic advantage of intratumoral peptide injection as an option for antigen-specific cancer immunotherapy

**Fig. 4:**
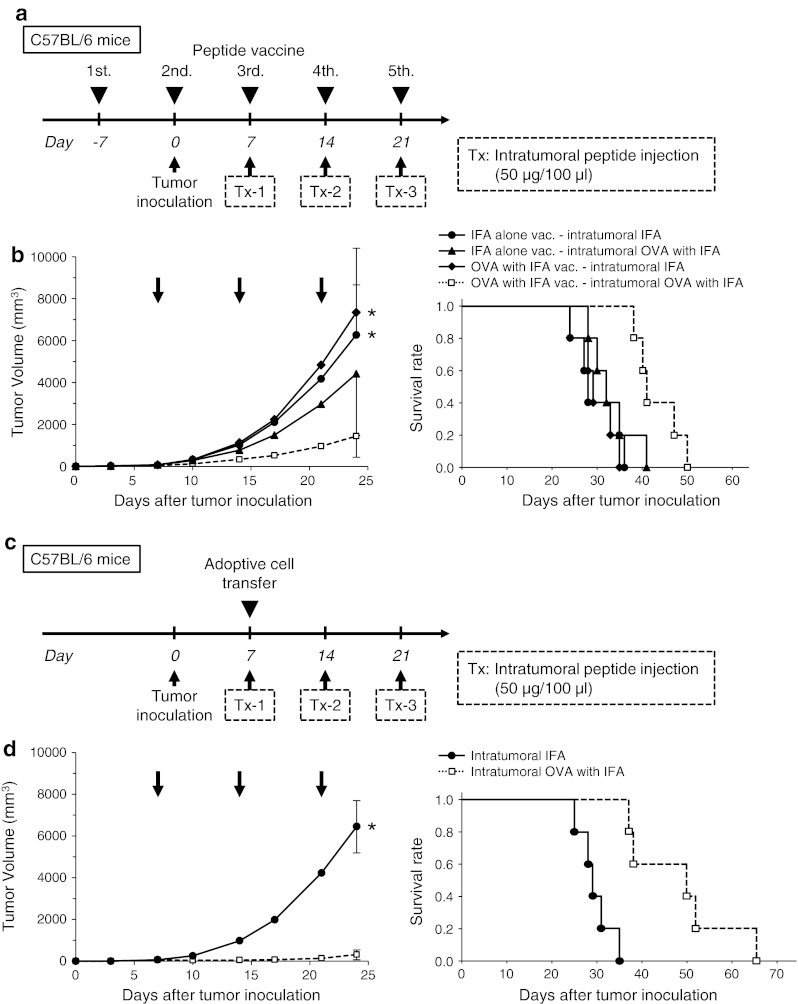

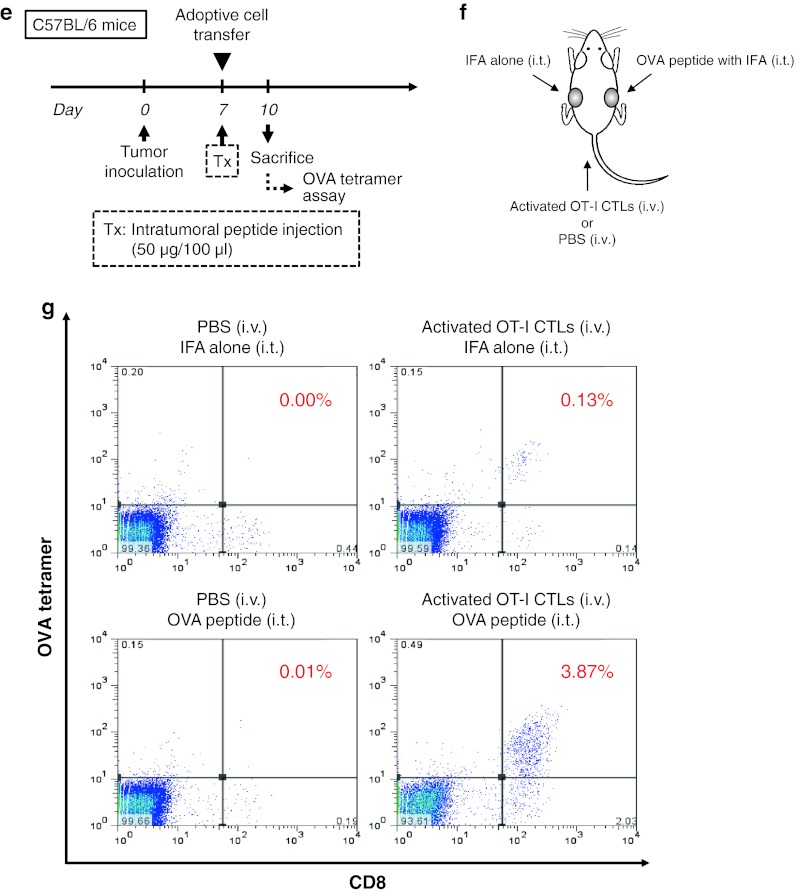

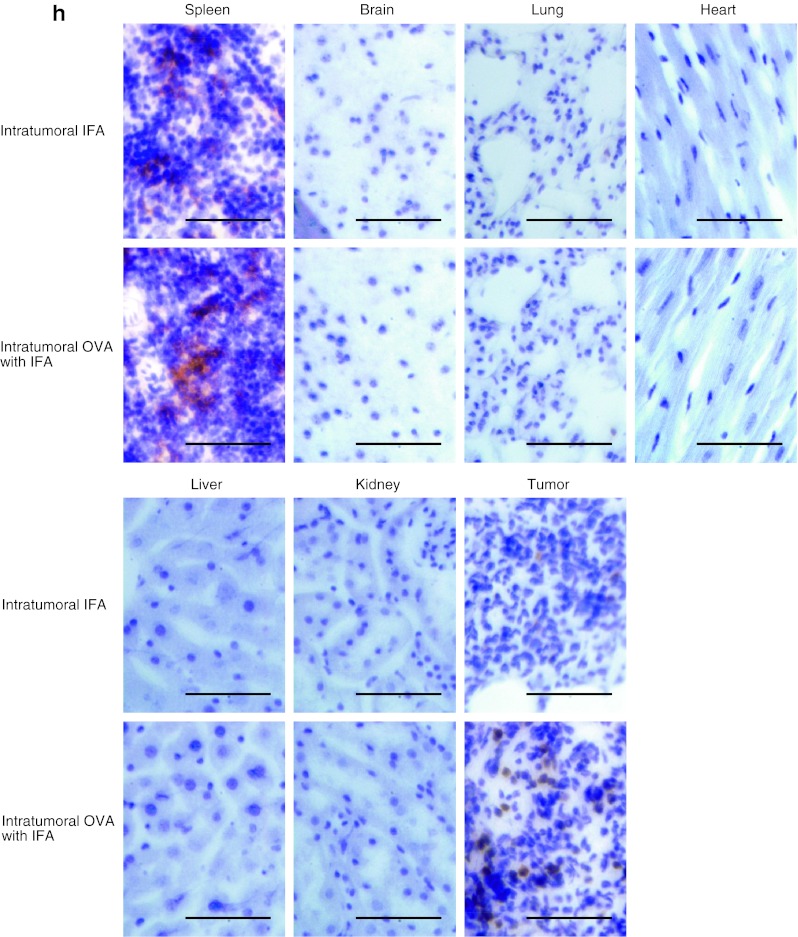
Therapeutic advantage of intratumoral peptide injection as an option for antigen-specific cancer immunotherapy. (**a** and **b**) Peptide vaccine model. (**c** and **d**) Adoptive cell transfer model. (**a** and **c**) Treatment schedule. (**b** and **d**) Tumor growth and Kaplan–Meier survival *curves*. Tumor growth was expressed by mean tumor volume; *bars* (SD). *P < 0.05 compared with the treatment group (Mann–Whitney U test). The survival of mice in the treatment group was significantly better than that in the control groups (*P* < 0.05) (log-rank test). Five mice were used in each group. **e** Schedule for analysis of local accumulation of OVA-specific CTLs in an adoptive cell transfer model. **f** Experimental schematic representation. Two tumors were implanted per mouse (5 × 10^4^ cells/100 μl). One tumor was injected with the OVA peptide plus IFA, and the other with IFA alone. **g** OVA tetramer assay. Local accumulation of OVA-specific CTLs was confirmed in a tumor injected with the OVA peptide plus IFA. Data are representative of three independent experiments. **h** Immunohistochemical staining of CD8 in tumor and normal tissues. Spleen was used as positive control. *Scale bars*, 50 μm

After the induction of OVA_257–264_ peptide-specific CTLs by peptide vaccination (Fig. 4a) or after the adoptive transfer of OVA_257–264_ peptide-specific CTLs (Fig. 4c), intratumoral injection of OVA_257–264_ peptide was effective against RMA cells, which are OVA-negative tumor cells. The RMA tumors cells that were injected intratumorally with OVA_257–264_ peptide demonstrated significant tumor growth inhibition, compared with mice without intratumoral injection of OVA_257–264_ peptide (*P* < 0.05). The survival rate in the treatment group was significantly better than that in the control groups (*P* < 0.05) (Fig. 4b, d). The group that did not receive OVA_257–264_ peptide vaccine but that received intratumoral peptide injection showed a partial treatment effect (Fig. 4b).

To obtain direct evidence that intratumoral peptide injection leads to local accumulation of antigen-specific CTLs, an OVA tetramer assay was performed using an adoptive cell transfer model (Fig. 4e). Two RMA tumors were bilaterally implanted per mouse. One tumor was injected with the OVA_257–264_ peptide plus IFA, and the other tumor with IFA alone (Fig. 4f). As shown in Fig. 4g, the tumor that underwent both adoptive cell transfer of activated OT-I CTLs and intratumoral injection of the OVA peptide contained more OVA-specific CTLs than the other tumors. Local accumulation of OVA-specific CTLs after intratumoral injection of the OVA_257–264_ peptide was confirmed by OVA tetramer assay.

Neither toxic signs nor death due to intratumoral injection of the OVA_257–264_ peptide was observed. Moreover, to evaluate the risk of autoaggression by intratumoral peptide injection, the tissues of treated mice in an adoptive cell transfer model were pathologically examined. The spleen, brain, lung, heart, liver, kidney, and tumor were critically scrutinized, and the findings were compared with those from mice that had intratumoral injection with IFA alone. In mice treated with intratumoral injection of OVA_257–264_ peptide, a larger number of CD8^+^ T-cells had infiltrated the RMA tumor 24 days after the transfer of OT-I CTLs and 10 days after the last intratumoral injection of OVA_257–264_ peptide. However, the simultaneous infiltration of normal tissues by CD8^+^ T-cells was not observed (Fig. 4h). These results suggest that peptide from intratumoral injection did not spread into normal tissues.

### The effect of antigen spreading to another tumor after intratumoral peptide injection

**Fig. 5:**
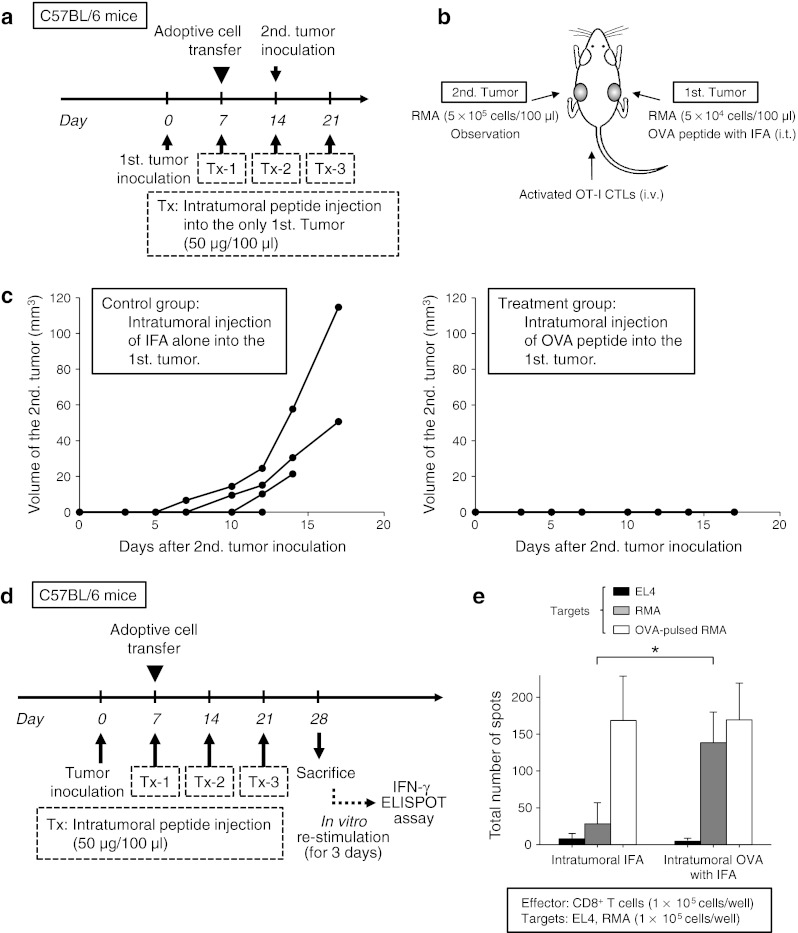
Effect of antigen-spreading to another tumor after intratumoral peptide injection. **a** The schedule for the experiment on antigen-spreading effect in an adoptive cell transfer model. **b** Experimental schematic representation. Two tumors were metachronously implanted per mouse (first tumor: 5 × 10^4^ cells/100 μl, second tumor: 5 × 10^5^ cells/100 μl), and only the first tumor (*right back*) received intratumoral peptide injection. The second tumor (*left back*) was not treated, but was observed. **c** The growth of the second inoculated RMA tumor. *Four lines* indicate the tumor growth of each mouse. All four mice in the treatment group completely rejected the second tumor challenge. **d** The experiment schedule to confirm antigen spreading. **e** IFN-γ ELISPOT assay. EL4 cells were used as negative control targets. The data are expressed as mean values of three mice (SD). *P < 0.05 compared with control (Mann–Whitney U test)

Using an adoptive cell transfer model, we assessed the possibility of antigen-spreading effect after intratumoral peptide injection, as depicted in Fig. 5a. Two RMA tumors were bilaterally and metachronously implanted per mouse, and only the first tumors received intratumoral injection of the OVA_257–264_ peptide. The sizes of the second tumors were compared with those from mice that received intratumoral injection of IFA alone (Fig. 5b). Whereas the second tumors were established 14 days after the second tumor inoculation in three out of four control mice, all four peptide-loaded mice that had received intratumoral OVA_257–264_ peptide injection into their first tumor completely rejected the challenge of the second tumor, which did not receive intratumoral OVA_257–264_ peptide injection itself (Fig. 5c).

To confirm the hypothesis of antigen spreading, an IFN-γ ELISPOT assay was performed. RMA tumor-bearing C57BL/6 mice that had received adoptive transfer of OT-I CTLs and intratumoral injection of OVA_257–264_ peptide were killed, and their spleens were obtained 21 days after adoptive transfer and 7 days after the last intratumoral injection. CD8^+^ T-cells, isolated from the spleen cells using anti-CD8a magnetic beads, were incubated with irradiated RMA cells for 3 days. CD8^+^ T-cells were separated from RMA cells using anti-CD8a magnetic beads before the assay. An IFN-γ ELISPOT assay was performed in duplicate using CD8^+^ T-cells as effector cells and RMA cells as target cells (Fig. 5d). The mice that had received intratumoral injection of OVA_257–264_ peptide showed a significant response to OVA-negative RMA tumor cells compared with control mice that had received intratumoral injection of IFA alone (*P* < 0.05). The observed induction of RMA-derived antigen-specific CTLs provides evidence that antigen spreading occurred by treatment with intratumoral OVA_257–264_ peptide and intravenous OT-I CTLs (Fig. 5e).

## Discussion

We demonstrated that intratumoral peptide injection leads to additional peptide loading onto MHC class I molecules of tumor cells, causing enhanced CTL recognition of tumor cells. It is likely that a larger number of antigen-specific CTLs infiltrate the tumors after this procedure, and tumor cells are killed more easily because CTL activity depends on the peptide density of tumor cells in an HLA class I-restricted manner. In other words, intratumoral peptide injection enhances the antigenicity of tumor cells, regardless of whether the tumor cells originally expressed the antigen. To the best of our knowledge, this is the first study to show the efficacy of intratumoral peptide injection in detail. A previous report demonstrated that peptide injection around a tumor assisted the activity of low-avidity CTLs in an immunodeficient mouse model [21]. In addition, we demonstrated the advantage as a therapeutic modality combined with antigen-specific cancer immunotherapy without any adverse reactions associated with this procedure in mice. Intratumoral peptide injection can strengthen the efficacy of every kind of antigen-specific cancer immunotherapy and may be a useful therapeutic option.

This is the first study to describe anticancer treatment with CMV-derived peptide-specific CTLs. Virus-derived antigens, which are exogenous antigens, usually have stronger antigenicity than tumor-associated autoantigens. Therefore, virus-derived antigen-specific CTLs are easier to induce [22]. Theoretically, every kind of antigen is applicable to our procedure unless it is expressed in healthy human cells. However, it is unclear whether post-CMV-infected lesions are safe from CMV-specific CTL cytotoxicity. Further investigations are necessary regarding the possible clinical use of exogenous antigens, such as CMV-derived peptides.

We used NaHCO_3_, which is known to have therapeutic effects against tumors [23, 24], as a peptide diluent. However, our data demonstrated the efficacy of intratumoral peptide injection, because control animals which underwent intratumoral injection of IFA alone or IFA plus an irrelevant peptide also received NaHCO_3_.

In an in vivo tumor growth inhibition assay using a peptide vaccine model, the group that did not receive the OVA_257–264_ peptide vaccine but that received intratumoral peptide injections showed a partial treatment effect. This indicates that intratumoral or peritumoral antigen-presenting cells recognized intratumorally injected OVA_257–264_ peptide and induced OVA_257–264_ peptide-specific CTLs after three intratumoral peptide injections. However, we showed in this study that intratumoral peptide injection attracted more OVA_257–264_ peptide-specific CTLs and was more effective when combined with peptide vaccines or adoptive cell transfer therapies.

A limitation of intratumoral peptide injection is its delivery method. First, immunotherapy is expected to contribute toward cancer therapy especially in the early stages or in the prevention of recurrence, in which cancer sites, the so-called “micro lesions,” are undetectable by imaging modalities. However, intratumoral peptide injection must be limited to the tumors, which are detectable by imaging modalities, and can be approached with a needle. Second, it is difficult to spread the peptides over the whole tumor by intratumoral injection, especially against large tumors. Moreover, it is difficult to approach all of the multiple tumors. This procedure might limit the ability of immunotherapy as a systemic therapy. If a novel method of delivering peptides to tumor cells selectively through a systemic route is established in the future due to advances in drug-delivery technologies, this method will become more suitable for clinical application.

Another limitation is that it requires the presence of MHC class I molecules. The potential loss of MHC class I expression in tumors would lead theoretically to the failure of this approach. Previous reports have indicated that 61–85 % of breast cancers had loss of or decreased HLA class I expression [25–27]. On the other hand, the down-regulation of HLA class I was less frequently observed in other cancers [27–30]. Before clinical application, it is necessary to select cancers in which HLA class I expression is sufficiently high.

Antigen-spreading effects have been observed following anticancer immunotherapy [31–34]. The second tumor challenge is easily rejected due to immunological memory. Therefore, we fixed the number of implanted tumor cells as the second tumors could be established. In this study, we report evidence of an antigen-spreading effect after intratumoral peptide injection. If this antigen-spreading effect is sufficiently steady and reliable, intratumoral peptide injection may even be effective against imaging-invisible or unapproachable tumors.

**Fig. 6:**
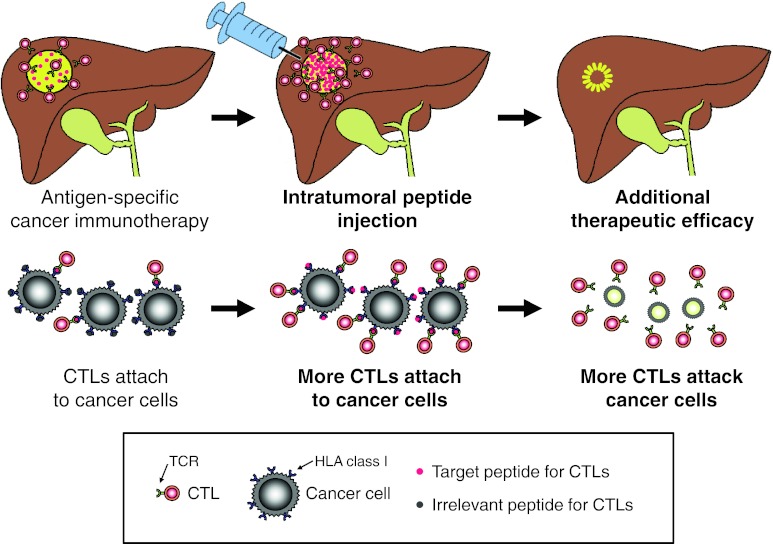
A proposed mechanistic model of intratumoral peptide injection for improvement in antigen-specific cancer immunotherapy of solid tumors

In conclusion, intratumoral peptide injection is an attractive strategy for enhancing tumor cell antigenicity. It can induce additional peptide loading onto tumor cells, making tumor cells more antigenic for antigen-specific CTL activity against tumor cells. Moreover, it may be a useful option for improvement in antigen-specific cancer immunotherapy against solid tumors (Fig. 6).

## Abbreviations

CTL: Cytotoxic T lymphocyte
HLA: Human leukocyte antigen
GPC3: Glypican-3
HCC: Hepatocellular carcinoma
MHC: Major histocompatibility complex
CMV: Cytomegalovirus
HIV: Human immunodeficiency virus
OVA: Ovalbumin
TAP: Transporter associated with antigen processing
FBS: Fetal bovine serum
IFN: Interferon
ELISPOT: Enzyme-linked immunospot
IFA: Incomplete Freund’s adjuvant
PBMC: Peripheral blood mononuclear cell
